# Rational Function-Based Approach for Integrating Tableting Reduced-Order Models with Upstream Unit Operations: Lubricants and Glidants Case Study

**DOI:** 10.3390/ph18101514

**Published:** 2025-10-09

**Authors:** Sunidhi Bachawala, Dominik Tomasz Nasilowski, Marcial Gonzalez

**Affiliations:** 1 School of Mechanical Engineering, Purdue University, West Lafayette, IN 47907, USA; 2 Ray W. Herrick Laboratories, Purdue University, West Lafayette, IN 47907, USA

**Keywords:** tableting, reduced-order models, bivariate rational functions, glidants, lubricants

## Abstract

**Background/Objectives**: Glidants and lubricants are commonly used pharmaceutical excipients that enhance powder flowability and reduce inter-particle friction, respectively, but they also negatively impact critical quality attributes such as tablet tensile strength and drug release rate. Quantifying these effects is essential as the pharmaceutical industry transitions from batch to continuous manufacturing. **Methods**: This study develops a rational-function-based modeling approach to capture the effects of lubricants and glidants on tableting. The framework automatically identifies upstream critical material attributes and process parameters, such as excipient concentration and mixing time, and describes their coupling to first and second orders. Reduced-order models were constructed to evaluate the influence of these variables on the four stages of powder compaction—die filling, compaction, unloading, and ejection—using formulations composed of 10% acetaminophen, microcrystalline cellulose, and varying small concentrations of magnesium stearate or colloidal silica. Tablets were fabricated across a wide range of relative densities by varying dosing position and turret speed. **Results**: The modeling approach successfully quantified the effects of lubricant and glidant mixing conditions on each compaction stage, providing mechanistic insight into how upstream conditions propagate through the tableting process and influence critical quality attributes. **Conclusions**: Overall, the rational-function-based framework offers a systematic approach to quantify and predict the impact of lubricants and glidants on tablet performance, thereby enhancing product and process understanding in continuous manufacturing.

## 1. Introduction

Magnesium stearate (MgSt) is a lubricant used in most formulations of pharmaceutical solid-dosage forms. It is typically present as fine nanoparticles, which coat microsized excipient and active pharmaceutical ingredient particles (APIs) [1]. Lubrication is an important unit operation in pharmaceutical powder processing. Mixing of small quantities of MgSt (0–2% *w*/*w*) with the formulation blend reduces friction between particles and along die walls [2,3] However, some blends are prone to over-lubrication, i.e., to a significant reduction in tablet tensile strength due to the inter-particle solid bridges being substantially weakened by the MgSt coating [4,5]. It is worth noting that tableting of brittle materials tends to be mostly insensitive to lubrication since unlubricated surfaces are generated during fragmentation. In contrast, materials that undergo plastic deformation without fragmentation, such as microcrystalline cellulose (MCC), exhibit sensitivity to lubrication [2,6,7].

Although the aforementioned effect of lubricants on particle interactions facilitates tableting by reducing the compaction force, ejection force, and heat generated, it also degrades tablet critical quality attributes (CQAs). Specifically, an addition of lubricants to pharmaceutical blends reduces tablet tensile strength, increases bulk density, and slows down drug release due to induced hydrophobicity [8,9]. Razavi et al. [10] have proposed quantitative models to describe the effects of lubricant concentration and mixing time on the tensile strength and elastic modulus of tablets. However, less research has been done to quantify the effects of the lubricant on all the tablet CQAs and critical process parameters (CPPs) of the manufacturing process.

Similarly, colloidal silica (CS) is a glidant used in small quantities (0–0.5% *w*/*w*) in most formulations to improve the flowability of the pharmaceutical blend [11]. In continuous oral solid dosage manufacturing, material flow is facilitated primarily by gravity and, hence, powder must inherently be easily flowable. Pingali et al. [9] showed evidence that adding CS to blends containing MgSt reduces the drug release rate even further. The authors also demonstrated that these effects are regulated by the amount of shear strain imparted during mixing, or under controlled conditions using a shear cell processor, and by the mixing order in which the blend is prepared [1]. Hentzschel et al. [12] showed that plastic deformation of all powder blends decreased with increasing silicate concentration. However, little research has been carried out to quantify the effects of the glidant on all the tablet CQAs and critical process parameters (CPPs) of the manufacturing process.

The quantification of lubricant and glidant effects becomes of paramount importance as the pharmaceutical industry transitions from batch manufacturing to continuous manufacturing, and as it follows the Quality by Design (QbD) framework for product and process development. This QbD paradigm was introduced by the U.S. Food and Drug Administration (FDA) in 2004, and it ensures product quality by design rather than by inspection. It mitigates risks and reduces the likelihood of defects or manufacturing deviations [13,14,15]. Complementing this paradigm is the concept of Quality by Control (QbC), which also underscores the importance of real-time monitoring and control of critical process parameters in continuous manufacturing [15,16]. Real-time control of tablet CQAs can be achieved by using the predictive models for CQAs and CPPs in the implementation of a nonnlinear model predictive control strategy [16].

Data driven modeling is a common approach used in the industry to model the tableting process. Wang et al. [17] developed a partial least squares (PLS) model to predict tablet CQAs, such as ejection force, breaking force, and disintegration time, using MgSt material properties (e.g., composition, particle size, bulk density, etc.) and its concentration as inputs. Mareczek et al. [18] used PLS and principal components analysis (PCA) to develop models of tablet tensile strength using 119 material descriptors, including conditioned bulk density, packing fraction, and particle size distribution parameters. The results of PLS and PCA analyses elucidated previously unknown relationships between material properties and tablet tensile strength. Finally, Deebes et al. [19] developed a data-driven plant-wide model using a combination of random forest regressors and gradient boosting machines to model each stage of the pharmaceutical plant, as well as by using Gaussian mixture models for error characterization and uncertainty reduction. A more detailed review of multivariate modeling techniques for tablet manufacturing, including data-driven methods, is available in [20] and references therein. Despite their success, the primary challenges faced by data-driven models include limited generalizability, lack of interpretability, and the need for large datasets for parameter estimation.

Reduced-order models (ROMs) are an essential cornerstone for realizing real-time monitoring and control of unit operations. These models result from a trade-off between complexity and performance, but they are still based on product and process understanding [21]. Hence, to support the QbC paradigm and the adoption of continuous manufacturing operations, the aim of this work is to develop tableting ROMs to quantitatively predict lubricant and glidant effects on process CPPs and tablet CQAs. Specifically, we propose to use the rational function-based approach proposed by Bachawala et al. [22] to systematically and automatically integrate lubricant and glidant mixing with tableting. The approach systematically identifies the upstream critical material attributes and process parameters that describe the coupling, e.g., the glidant and lubricant concentration and the total shear imparted to the blend during mixing. The aforementioned trade-off between model complexity (i.e., the number of model parameters) and the goodness of the model prediction (i.e., the sum of squared errors) is controlled during model selection and parameter identification by using the Akaike Information Criterion. The integration of lubricant and glidant mixing with tableting is illustrated using a formulation composed of 10% by mass of acetaminophen, microcrystalline cellulose, and varying small concentrations of MgSt or CS. Tablets are fabricated using a Natoli NP-400 (Natoli Engineering Company, Inc., St. Charles, MO, USA) tablet press.

In this work, we make the following novel contributions:

1. We apply the rational function framework to design ROMs to model the effect of lubricants and glidants on tableting.
2. We expand the rational function library used in Bachawala et al. [23] to include rational functions of the second degree to enable the modeling of nonmonotonic behavior in arbitrary directions.
3. We analyze the limiting behavior of general rational functions and enforce mechanistic constraints on the parameters of rational functions.
4. Unlike the full-factorial design of experiments used in Bachawala et al. [23], we use a Latin hypercube experimental design to control the number of experiments while covering the design space.

This paper is organized as follows. Section 2.1 outlines the experimental campaigns for the formulations with magnesium stearate and with colloidal silica. This section also describes the acquisition of the table quality properties and process parameters. Section 2.2 presents the tableting ROMs used in this work, and Section 2.3 describes the integration of these ROMs with the lubricant and glidant blending unit operations using the rational function-based approach proposed by Bachawala et al. [23]. The model library used in this paper is enhanced with a complementary family of normalized bivariate rational functions. The mathematical form of the optimal selected ROMs, their parameters, and the mechanistic insights that emerge from them are presented in Section 3. The effects of lubricant and glidant on tableting CPP and CQA, and the application of these ROMs in real-time control to enable continuous pharmaceutical manufacturing, are discussed in Section 4. Finally, conclusions and areas for improvement are discussed in Section 5.

## 2. Materials and Methods

### 2.1. Preparation and Characterization of Tablets

Experimental campaigns were conducted to elucidate the lubricant and glidant effects on process CPPs and tablet CQAs. The powder blends contained microcrystalline cellulose Avicel PH200 (MCC) from IMCD Group US (Westlake, OH, USA), 10% *w*/*w* acetaminophen (APAP) from Mallinckrodt Pharmaceuticals (Dublin, Ireland), and varying concentrations of MgSt or CS (0–2% *w*/*w*, or 0–0.2% *w*/*w*, respectively). One-kilogram blends with MgSt (Spectrum Chemical, Gardena, CA, USA) were mixed in a 5 L Tote blender (Tote Systems International, Fort Worth, TX, USA) for 11–30 min, and one-kilogram blends with CS (Cabot Corporation, Tuscula, IL, USA) were mixed for 10–30 min. Although practical mixing times may be shorter than 20 min, experimental data were collected up to 30 min to model the potential effects of over-lubrication. Tablets were fabricated using a Natoli-NP400 tablet press and D-type tooling, with a shallow cup depth of h=0.3302 mm and an in-die thickness of 2.44 mm. A dosing position ranging from 9 to 13 mm and a turret speed varying from 11 to 22 rpm were used for blends with MgSt, and a dosing position ranging from 7 to 11 mm and a turret speed varying from 25 to 35 rpm were used for blends with CS.

The MATLAB R2021a function lhsdesign was used to generate a Latin hypercube design [24] that samples the turret speed, dosing position, MgSt/CS concentration, and mixing time. In this work, since there are four input variables, the full factorial experimental design used in prior work [22] is infeasible. In order to maximize the design space coverage while controlling the number of experiments, we chose the Latin Hypercube design for the design of experiment, which is a statistical technique used to generate samples from multiple input variables while ensuring good coverage of the entire design space. The experimental campaigns in this work comprised 20 experiments for blends with MgSt, and of 30 experiments for blends with CS. The bulk density of each one-kilogram blend was determined using a half-kilogram sample, which was divided into eight equal parts using a Retsch PT100 spin riffler (RETSCH GmbH, Haan, Germany) to measure the weight and volume of each sub-sample.

**Figure 1:**
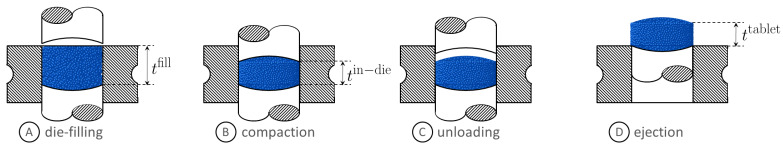
Stages of tablet compaction. Image courtesy of Gonzalez [26].

At the start of each experimental run, the tablet press hopper was filled with 0.5 kg of the blend to ensure enough powder was available to maintain steady-state conditions. During each run, a SOTAX AT4 tablet tester (SOTAX Corp, Westborough, MA, USA) was used to measure the thickness, diameter, weight, and hardness of 50 tablets under steady-state manufacturing conditions. Using these measurements, the pre-compression ($\rho^{\mathrm{pc}}$), main-compression ($\rho^{\mathrm{in}-\mathrm{die}}$), and after-ejection ($\rho^{\mathrm{tablet}}$) tablet relative densities were calculated following Bachawala et al. [23]. Specifically, for a given filling weight *W* and a true density $\rho_{t}$ of the blend, these relative densities are given by

(1) $$\rho^{\mathrm{pc}}\left|\mathrm{in}-\mathrm{die}\right|\mathrm{tablet}=\frac{W}{\rho_{t}\;V^{\mathrm{pc}}\left|\mathrm{in}-\mathrm{die}\right|\mathrm{tablet}},$$

where the corresponding tablet volumes are given by

(2) $$V^{\mathrm{pc}|\mathrm{in}-\mathrm{die}|\mathrm{tablet}}=\frac{\pi D^{2}t^{\mathrm{pc}|\mathrm{in}-\mathrm{die}|\mathrm{tablet}}}{4}+\frac{\pi h}{6}\left(\frac{3D^{2}}{4}+h^{2}\right)$$

with D=8 mm being the die diameter, and $t^{\mathrm{pc}|\mathrm{in}-\mathrm{die}|\mathrm{tablet}}$ the corresponding thicknesses (see Figure 1). The tensile strength $\sigma_{t}$ for the convex tablets is obtained from Pitt’s model [25] using the measured hardness *F*, i.e.,

(3) $$\sigma_{t}=\frac{10F}{\pi D^{2}\left[2.84\left(H^{\mathrm{tablet}}/D\right)0.126\left(H^{\mathrm{tablet}}/t^{\mathrm{tablet}}\right)+3.15\left(t^{\mathrm{tablet}}/D\right)+0.01\right]},$$

with $H^{\mathrm{tablet}}=t^{\mathrm{tablet}}+2h$. It bears emphasis that not all measurements are available on-line to implement real-time control under the QbC paradigm [16].

### 2.2. Tableting Reduced-Order Models

Tableting consists of four main stages, namely die-filling, pre-compression, main-compression, and tablet ejection. Powders are first filled into a die cavity which is confined on the bottom by a punch tip (see schematic A in Figure 1). During pre-compression, a relatively small force is exerted by the upper punch to remove air and prevent capping of the tablet during ejection. In the main-compaction stage, a larger force is applied on the powder bed to reach the desired in-die tablet thickness (see schematic B in Figure 1). The tablet undergoes elastic recovery during the unloading and ejection stages (see schematics C and D in Figure 1). It is worth noting that the axially compacted powder creates a residual stress in the radial direction and, in turn, an ejection force needs to be exerted by the bottom punch in order to overcome tablet/die friction.

The development of mechanistic and semi-mechanistic reduced-order models (ROMs), resulting from a trade-off between complexity and performance, but still based on product and process understanding, forms an essential cornerstone for process design, optimization, and control in pharmaceutical manufacturing. Here, we adopt semi-mechanistic formulae which are in remarkable agreement with the particle-level calculations that use the particle mechanics approach for modeling the consolidation and compaction of powders under large deformations [26]. These semi-mechanistic ROMs have also been used by Bachawala et al. [23] to integrate tableting with dry granulation. Specifically, mathematical expressions for the die-filling efficacy coefficient (η), the Kawakita’s compaction force $\left(F_{\mathrm{punch}}\right)$, the elastic recovery $\left(\epsilon_{\rho }\right)$, the tablet density $\left(\rho^{\mathrm{tablet}}\right)$, and the Leunberger’s tablet tensile strength $\left(\sigma_{t}\right)$ are listed below.

(4) $$\begin{array}{cc} \eta & =\frac{W}{\rho_{b}V_{\mathrm{fill}}} \end{array}$$

(5) $$\begin{array}{cc} F_{\mathrm{punch}} & =\frac{\pi D^{2}\left(\rho^{\mathrm{in}-\mathrm{die}}-\rho_{c}\right)}{4b\left(\rho^{\mathrm{in}-\mathrm{die}}\left(a-1\right)+\rho_{c}\right)} \end{array}$$

(6) $$\begin{array}{cc} \epsilon_{\rho } & =\epsilon_{0}{\left(\frac{\rho^{\mathrm{in}-\mathrm{die}}-\rho_{c,\epsilon }}{1-\rho_{c,\epsilon }}\right)}^{n} \end{array}$$

(7) $$\begin{array}{cc} \rho^{\mathrm{tablet}} & =\rho^{\mathrm{in}-\mathrm{die}}\left(1-\epsilon_{\rho }\right) \end{array}$$

(8) $$\begin{array}{cc} \sigma_{t} & =\sigma_{0}\left[1-\left(\frac{1-\rho^{\mathrm{tablet}}}{1-\rho_{c,\sigma_{t}}}\right)e^{\left(\rho^{\mathrm{tablet}}-\rho_{c,\sigma_{t}}\right)}\right] \end{array}$$

The model parameters have the following mechanistic interpretation:

- The filling efficacy coefficient is typically less than one due to the tablet press inefficiencies in the feed-frame, turret and die-filling dynamics; that is, the tablet weight *W* is typically less than the bulk density $\rho_{b}$ times the fill volume of the die $V_{\mathrm{fill}}$—which is given by
  (9) $$V^{\mathrm{fill}}=\frac{\pi D^{2}}{4}t^{\mathrm{fill}}+\frac{\pi h}{6}\left(\frac{3D^{2}}{4}+h^{2}\right)$$
  with *h* being the cup depth.
- The Kawakita compaction force equation was adapted by Bachawala and Gonzalez [27] to depend on the in-die tablet relative density $\rho^{\mathrm{in}-\mathrm{die}}$, and the critical in-die relative density $\rho_{c}$ at which jamming occurs and particle-level deformation begins. The parameters *a* and 1/b [MPa] represent the total degree of compression or total compressibility of the powder, and the compaction pressure required to reach half of the maximum volume reduction a/2 (i.e., the pressure at a relative in-die density such that $\left(\rho^{\mathrm{in}-\mathrm{die}}-\rho_{c}\right)/\rho^{\mathrm{in}-\mathrm{die}}=a/2$), respectively [28,29,30].
- The tablet undergoes elastic recovery during the unloading and ejection stages, and $\epsilon_{0}$ corresponds to the elastic recovery at full compaction (or zero porosity), while $\rho_{c,\epsilon }$ is the smallest in-die tablet relative density at which elastic recovery is observed. The exponent *n* accommodates for nonlinearities and generalizes the otherwise linear trend [23].
- The tablet tensile strength depends on the tablet relative density $\rho^{\mathrm{tablet}}$ after elastic recovery, the tensile strength $\sigma_{0}$ of a tablet with a relative density of one (or zero porosity), and the smallest tablet relative density $\rho_{c,\sigma_{t}}$ at which a tablet with adequate strength is formed [31].

These model parameters are next endowed with dependency on lubricant or glidant mixing conditions.

### 2.3. Rational Function-Based Approach for the Integration of Unit Operations

Multivariate rational functions have been used as surrogate models to successfully interpolate and extrapolate sparse data in many applications [32,33]. Bachawala et al. [22] have proposed a rational function-based approach to systematically and automatically integrate tableting ROMs with upstream unit operations. We adopted this approach to integrate lubricant and glidant mixing with tableting. Therefore, by following the same procedure, we identified the upstream CPP|CMA that describes the coupling, e.g., the glidant $c_{\mathrm{CS}}$ or lubricant $c_{\mathrm{MgSt}}$ concentration and the total shear imparted to the blend during mixing. Specifically, the coupling is done solely through model parameters ξ of the tableting reduced-order model M(ξ) by means of a nonlinear function of upstream CPP|CMA and its parameters θ, that is,

(10) M(ξ)∘ξ(CPP|CMA;θ)→M(CPP|CMA;θ)≡M(θ).

Next, we normalized the upstream CPP|CMA to be in the range of [0,∞), as shown in Table 1. In this work, $c_{\mathrm{MgSt}}^{\max}=2\%$ *w*/*w* and $c_{\mathrm{CS}}^{\max}=0.2\%$ *w*/*w* are considered for pharmaceutical formulations. Furthermore, in the two experimental campaigns described above, mixing of each one-kilogram blend is done using the same 5 L Tote blender and, in turn, the total shear imparted to the blend $\gamma_{\mathrm{mix}}$ and the total mixing time $t_{\mathrm{mix}}$ are interchangeable upstream CPPs. In contrast, continuous blenders are used in continuous manufacturing operations and, in turn, the total shear imparted depends on the impeller speed, the incoming mass-flowrate, and the equipment design variables, such as the shaft angle, blade width, and weir height [34,35]. In this work, a maximum mixing time of $t_{\mathrm{mix}}^{\max}=60$ min is considered for normalization purposes.

Bachawala et al. [23] adopted the following normalized bivariate rational function f(X,Y) to systematically integrate the unit operations

(11) $$\xi \left(\mathrm{CPP}|\mathrm{CMA};\theta \right)\to f\left(X,Y\right)=\frac{p_{1}XY+p_{2}X+p_{3}Y+p_{4}}{q_{1}XY+q_{2}X+q_{3}Y+1},$$

such that $\theta =\{p_{i},q_{j},r_{X},r_{Y}\}$, with i={1,2,3,4},j={1,2,3}, are the rational function parameters to be estimated, in general. This simple and flexible bivariate rational function has a limiting behavior at the upper and lower bounds that can be easily interpreted physically, that is

(12) $$\begin{array}{cc} \; & f\left(0,0\right)=p_{4} \end{array}$$

(13) $$\begin{array}{cc} \; & f\left(0,+\infty \right)=p_{3}/q_{3} \end{array}$$

(14) $$\begin{array}{cc} \; & f\left(+\infty ,+\infty \right)=p_{1}/q_{1} \end{array}$$

(15) $$\begin{array}{cc} \; & f\left(+\infty ,0\right)=p_{2}/q_{2}. \end{array}$$

There are two distinct limits in the integration of lubricant and glidant mixing with tableting. First, if the blend does not contain any lubricant (glidant), then the tableting ROMs parameters (see Table 2) ought to be independent of mixing time. Second, if the blend contains lubricant (glidant) but it is not mixed, then the tableting ROMs parameters (see Table 2) ought to be independent of the lubricant (glidant) concentration. Furthermore, these two limits ought to be the same and equal to the case of 10% APAP and 90% MCC (i.e., $p_{2}/q_{2}=p_{3}/q_{3}=p_{4}$). Therefore, the generalized bivariate rational function used in the integration of lubricant and glidant mixing with tableting simplifies to

(16) $$\xi \left(\mathrm{CPP}|\mathrm{CMA};\theta \right)\to f\left(X,Y\right)=\frac{p_{1}XY+p_{4}\left(q_{2}X+q_{3}Y+1\right)}{q_{1}XY+q_{2}X+q_{3}Y+1}$$

One of the features of (16) is that the function is monotonic in *X* and *Y*. However, we observed that the bulk density of the blends with the lubricant had a nonmonotonic trend with respect to the lubricant concentration. Therefore, to capture these nonmonotonic trends in data, in this work, we propose extending the model library to include a different normalized bivariate rational function f(X,Y) and demonstrate that similar limiting behavior is recovered. Specifically, from the more general expression

(17) $$\xi \left(\mathrm{CPP}|\mathrm{CMA};\theta \right)\to f\left(X,Y\right)=\frac{p_{5}X^{2}+p_{6}Y^{2}+p_{1}XY+p_{2}X+p_{3}Y+p_{4}}{q_{5}X^{2}+q_{6}Y^{2}+q_{1}XY+q_{2}X+q_{3}Y+1}$$

the limiting behavior is enforced (i.e., $p_{6}/q_{6}=p_{5}/q_{5}=p_{4}$), and the linear terms are neglected to draw a distinction from (16), i.e.,

(18) $$\xi \left(\mathrm{CPP}|\mathrm{CMA};\theta \right)\to f\left(X,Y\right)=\frac{p_{1}XY+p_{4}\left(q_{5}X^{2}+q_{6}Y^{2}+1\right)}{q_{1}XY+q_{5}X^{2}+q_{6}Y^{2}+1}$$

Lastly, a model library F is built by progressively simplifying the bivariate rational functions (16) and (18) to fifteen models, as shown in Table 3. Furthermore, for simplicity and computational efficiency, the model library contains only 43 pairs (one for each model parameter in ξ; see Table 2) out of all 225 possible combinations (see Table 4). For example, in the tensile strength model, the parameters $\sigma_{0}$ and $\rho_{c,\sigma_{t}}$ are allowed twenty-five model combinations of the form (i,i),(i,9),(9,i), with model number i=1,2,…,15.

The final step of the rational function-based approach [23] is to choose the best model combination from the library F, i.e., the combination that provides accurate predictions with the least number of parameters ($N_{p}$). The prediction accuracy is typically measured by the sum of squared errors between the model prediction M(θ) and experimental data E. This trade-off between model complexity and accuracy is effectively captured by the Akaike Information Criterion (AIC) [36,37], defined as

(19) $$\mathrm{AIC}=\left\{\begin{array}{cc} n\ln\left(\frac{1}{n}\sum_{i=1}^{n}{\left(M^{i}\left(\theta \right)-E^{i}\right)}^{2}\right)+2N_{p}\; & n\geq 30\;\\ n\ln\left(\frac{1}{n}\sum_{i=1}^{n}{\left(M^{i}\left(\theta \right)-E^{i}\right)}^{2}\right)+2N_{p}+\frac{2N_{p}\left(N_{p}+1\right)}{n-N_{p}-1} & n<30 \end{array}\right.,$$

where *n* is the number of experimental data points. The model with the lowest AIC is the best. For small sample sizes, the AIC often leads to overfitting. To improve its performance, a second-order bias correction is introduced.

Model selection and parameter estimation are then automatically realized by solving a nested optimization problem [23], where the lower subproblem identifies the optimal model parameters for a given model by minimizing the sum of squared errors between the predicted and experimental data, and the upper subproblem selects the best model by minimizing the AIC. In this work, we use MATLAB’s genetic algorithm (ga), fmincon, and global search (GlobalSearch) in that specific order to solve a constrained nonlinear optimization problem and obtain optimal model parameters. This ensures that the estimated parameters are not biased to the initial guess and represent the global solution. The inequality constraints are given by $p_{i}>0,q_{j}>0$ with i=1,4,j=1,2,3,5,6, and r∈(0,10] are imposed to ensure the parameters ξ>0. The parameters $a,\epsilon_{0},\rho_{c},\rho_{c,\epsilon },\rho_{c,\sigma_{t}}$ represent relative quantities and must be in the range [0,1]. Furthermore, the onset of critical compaction, elastic recovery, and tablet relative densities occur consecutively, i.e., $\rho_{c,\epsilon }\in \left({\bar{\rho }}_{c},1\right)$, $\rho_{c,\sigma_{t}}\in \left({\bar{\rho }}_{c,\epsilon },1\right)$, with ${\bar{\rho }}_{c}$ and ${\bar{\rho }}_{c,\epsilon }$ being the lower bounds of the compaction and elastic recovery critical relative densities, respectively.

## 3. Results

The CMAs, CPPs, and CQAs measured and collected as part of the experimental campaign described in Section 2.1 were used to identify ROMs and their parameters for integrating lubricant or glidant mixing with tableting processes. Specifically, the models presented in Section 2.2 were adopted, and optimal model parameters were identified using the approach presented in Section 2.3.

In the following sections, the bulk density and tablet weight models for blends with MgSt and CS are discussed first. Later, the ROMs for each compaction, tablet density, and tensile strength are presented for the MgSt and CS blends. Finally, the effects of MgSt and CS on the tableting process are compared. These results are summarized in Table 5 and Table 6.

### 3.1. Bulk Density

The bulk density of the powder blend depends on the concentration of lubricant $c_{\mathrm{MgSt}}$ and glidant $c_{\mathrm{CS}}$ [8,39], as well as the mixing time or shear strain imparted to the system, with $\gamma_{\mathrm{mix}}\propto t_{\mathrm{mix}}$. It is experimentally observed that, as the total shear strain increases, the bulk density initially increases and ultimately reaches a plateau, during which no further change in the bulk density is observed [8]. We specifically propose that

(20) $$\rho_{b}=\phi \left(X,Y\right)\rho_{t}\left(c_{\mathrm{MgSt}}/\mathrm{CS}\right)$$

where $\rho_{t}\left(c_{\mathrm{MgSt}}/\mathrm{CS}\right)$ is the true density of the blend, ϕ(X,Y) is the packing fraction of the granular system, and the normalized variables *X* and *Y* depend on the lubricant (glidant) concentration and total mixing time (see Table 1). The generalized bivariate rational function presented in Equations (16) and (18) are appropriate for describing the packing fraction, since the same packing fraction $p_{4}\in \left[0,1\right]$ is expected for processing conditions where no lubricant (glidant) is added to the blend (i.e., X=0), as well as for no mixing conditions (i.e., Y=0). It is worth noting that the physical limiting behavior at large values of mixing time (here 60 min), together with the physical bounds for a packing fraction, enforce constraints on the model parameters, e.g., $0\leq p_{4}\leq p_{1}/q_{1}\leq 1$ for Equation (16).

**Figure 2:**
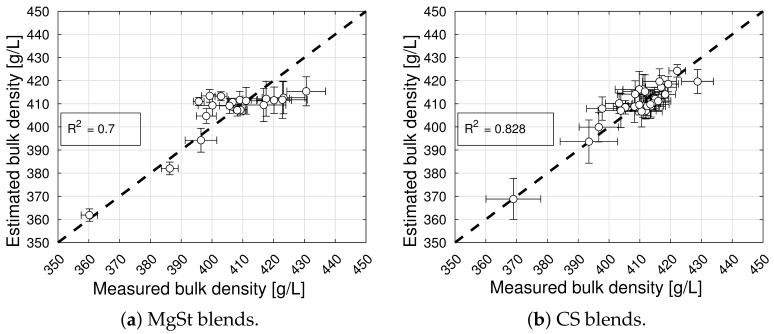
Goodness of bulk density prediction for blends with magnesium stearate (MgSt) and colloidal silica (CS).

**Figure 3:**
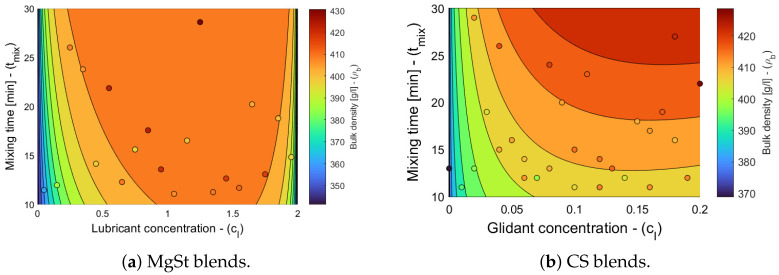
Bulk density contour plots for blends with magnesium stearate (MgSt) and colloidal silica (CS). The symbols overlaid on the contour plot represent bulk density measurements.

The model selection and parameter estimation approach proposed in Section 2.3 identifies model (13) for blends with magnesium stearate, and model (7) for blends with colloidal silica, as optimal within the model library F (see Table 7 and Table 8). It is interesting to note that the packing fraction of blends without a lubricant and without a glidant are predicted to be very similar (cf. $p_{4}=0.212$ in Table 5 and $p_{4}=0.244$ in Table 6) and close to the solid fraction of MCC (for commonly reported values of bulk density close to 0.3 g/cm^3^ and a true density close to 1.5 g/cm^3^), despite being obtained from two different datasets. This physical interpretability of the model parameters is one of the advantages of bivariate rational functions. Figure 2 shows the goodness of the bulk density models, and Figure 3 shows the bulk density predictions of the models and experimental values for the processing conditions described in Section 2.1. The model captures the experimentally observed trend of bulk density increasing with an increasing concentration and mixing time, and it shows that at a high mixing time, the bulk density reaches a plateau [8].

### 3.2. Tablet Weight

Tablet weight *W* is known to decrease with an increasing turret speed $n_{T}$, and with a decreasing filling height $t^{\mathrm{fill}}$ and paddle or feed frame speed $n_{F}$ (see, e.g., [40]). Furthermore, the tablet weight variability increases with an increasing bulk density and cohesion of the powder, with an increasing turret speed, and with a decreasing particle size [41]. The filling efficacy coefficient η is adopted in this work to describe these tablet press inefficiencies in the feed-frame, turret, and die-filling dynamics, and it is defined by Equation (4). Linear regression models of tablet weight and these CPP|CMA have been explored in the literature [40,41,42,43,44,45], but it is also known that the influence of higher-order terms is significant [42]. Therefore, in this work, we systematically explore higher-order models with linear and squared terms of the turret speed, feeder speed, and filling height.

To capture the behavior observed experimentally, Su et al. [46] have proposed that the weight is linearly proportional to the turret speed, with and without an additional linear proportionality to the filling height, that is

(21) $$\eta =\frac{W}{\rho_{b}V^{\mathrm{fill}}}=1-\xi_{1}\frac{n_{T}}{n_{F}}+\xi_{2}\frac{t^{\mathrm{fill}}}{D}$$

The limiting behavior of this relationship and its physical interpretation is discussed next. Specifically, if the production rate is given by

(22) $$\dot{m}=N_{d}n_{T}W=\eta \;N_{d}n_{T}\rho_{b}V^{\mathrm{fill}}$$

where $N_{d}$ is the number of die stations in the tablet press, then the simultaneous limit of the turret and feed frame speeds tending to zero must result in a zero production rate. Hence, it is straightforward to conclude that η can only be a function of $n_{F}/n_{T}$, while terms such as $n_{T}/n_{F}$ and $n_{F}/n_{T}$ of order two or higher are inadmissible. This observation leads to the following proposition for the filling efficacy coefficient with second-order terms

(23) $$\eta =\frac{W}{\rho_{b}V^{\mathrm{fill}}}=\xi_{1}+\xi_{2}\frac{n_{F}}{n_{T}}+\xi_{3}\frac{t^{\mathrm{fill}}}{D}+\xi_{4}{\left(\frac{t^{\mathrm{fill}}}{D}\right)}^{2}+\xi_{5}\frac{n_{F}}{n_{T}}\frac{t^{\mathrm{fill}}}{D}$$

**Figure 4:**
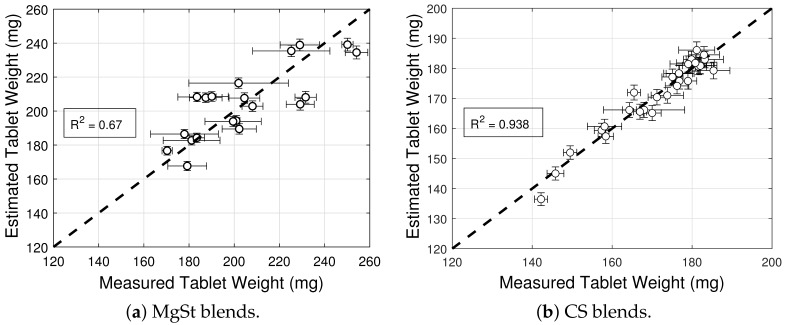
Goodness of tablet weight models prediction for blends with magnesium stearate (MgSt) and colloidal silica (CS).

**Figure 5:**
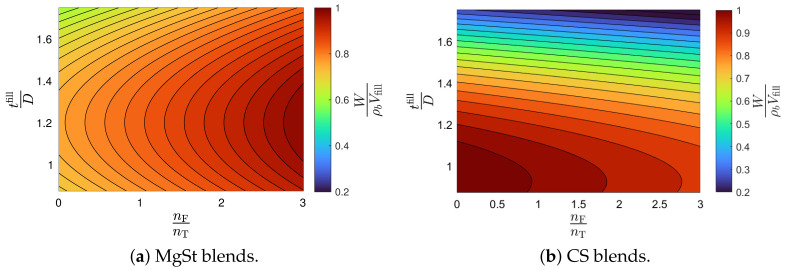
Filling efficacy contour plots for blends with magnesium stearate (MgSt) and colloidal silica (CS).

The model selection and parameter estimation approach proposed in Section 2.3 is here adapted to identify the optimal variations in general Equation (23). For completeness, Equation (21) is also included in the model library. Table 9 and Table 10 show that the model with $\xi_{1}=\xi_{5}=0$ is optimal for blends with magnesium stearate, whereas the model with $\xi_{5}=0$ for blends with colloidal silica. Figure 4 shows the goodness of the weight model, and Figure 5 shows contour plots of the filling efficacy for nominal tablet press operating conditions. It is evident from the figure that the filling efficacy decreases with an increasing $t^{\mathrm{fill}}/D$ for both the MgSt and CS blends, with the latter being more sensitive to changes in the filling height. The effect of $n_{F}/n_{T}$ is significantly less. These contour plots provide guidance for identifying operating conditions that maximize filling efficacy.

### 3.3. Compaction Force

**Figure 6:**
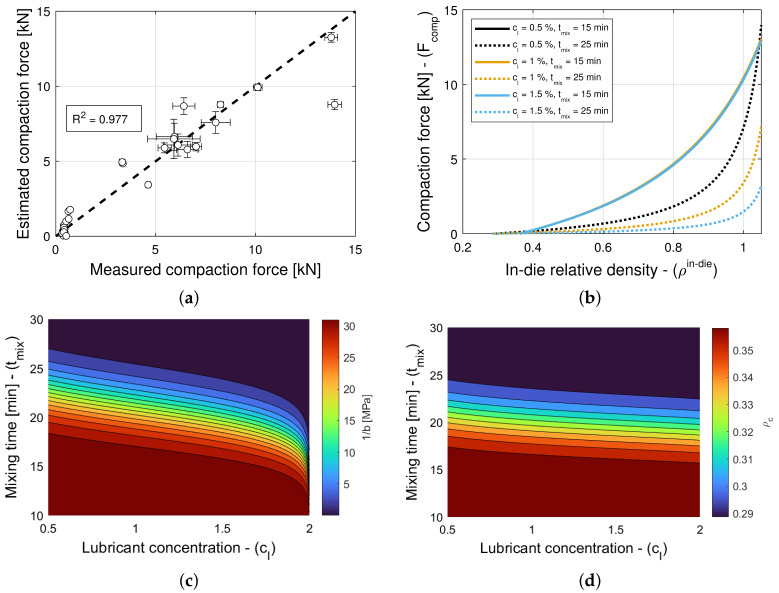
Compaction force for blends with magnesium stearate (MgSt). (**a**) Goodness of the compaction force prediction. (**b**) Predictions of the compaction force model. (**c**) Contour plot of 1/b. (**d**) Contour plot of $\rho_{c}$.

**Figure 7:**
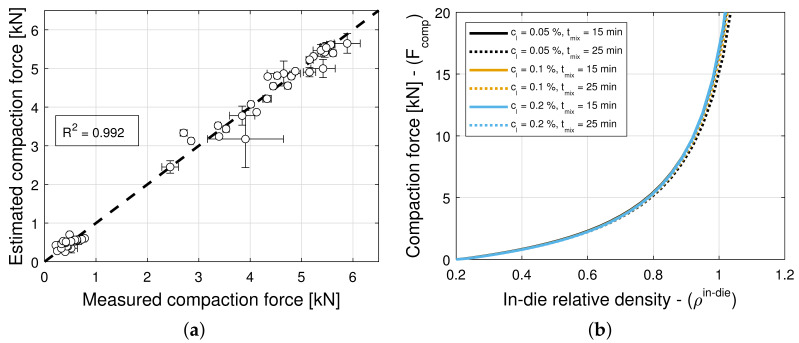
Compaction force for blends with colloidal silica (CS). (**a**) Goodness of the compaction force prediction. (**b**) Predictions of the compaction force model.

The addition of a lubricant or a glidant to a blend can have an impact on both Kawakita parameters, i.e., on *a* and 1/b in Equation (5), in general. Hence, the model selection and parameter estimation approach proposed in Section 2.3 is applied to a model library F that accommodates for a(X,Y) and $\rho_{c}\left(X,Y\right)$, with constant 1/b, as well as for 1/b(X,Y) and $\rho_{c}\left(X,Y\right)$, with constant *a*, while restricting attention to only variants of Equation (16) for simplicity. In turn, this systematic and automatic approach identifies models (9,3,3) and (6,9,6) as optimal for blends with magnesium stearate and colloidal silica, respectively (see Table 11 and Table 12). The details of each constrained variant of the normalized bivariate rational function are presented in Table 4, e.g., (9,3,3) is such that 1 parameter is used for *a*, 4 parameters are used for 1/b, 4 parameters are used for $\rho_{c}$, and $r_{X}$ and $r_{Y}$ are determined in (0,10]. Figure 6a and Figure 7a show scatter plots of the measured and estimated compaction force, demonstrating that the goodness of the compaction force prediction is high, with $R^{2}=0.977$ (Table 11) and $R^{2}=0.992$ (Table 12) for blends with magnesium stearate and colloidal silica, respectively. It is interesting to note for models with a very small number of parameters, as it is the case here, the Akaike Information Criterion might favor models that very closely fit the training data, i.e., $R^{2}\approx 1$, regardless of their simplicity. This is the case of the model selected for blends with colloidal silica, which is as good as model (9,9,9) with only three model parameters. The investigation and application of an AIC with higher-order estimates of information loss is a research direction worthwhile exploring (e.g., Equation (19) for n<30 is regarded as one using a second-order estimate of information loss).

Figure 6b and Figure 7b show predictions of the models for tablets formed with blends with different lubricant (glidant) concentrations and different mixing times. It is evident from Figure 6b that the compaction force is reduced with an increase in lubricant concentration or mixing time. In contrast, it is observed in Figure 7b that the compaction forces exhibits a negligible increase with an increase in glidant concentration and mixing time. This is in agreement with the observation that model (9,9,9) is nearly optimal for blends with colloidal silica, i.e., the compaction force is insensitive to the glidant in the blend, for concentrations under 0.2% *w*/*w*, achieving $R^{2}=0.988$. Hence, we select model (9,9,9) as optimal, since it has less parameters and explicitly does not depend on the glidant concentration.

The dependency of blends with magnesium stearate to the lubricant concentration and mixing time can be further investigated by studying contour plots of 1/b and $\rho_{c}$, as shown in Figure 6c and Figure 6d, respectively. The compaction pressure 1/b required to reach half of the maximum volume reduction a/2 [28,29,30] decreases as the lubricant concentration and mixing time increase. Similarly, the critical in-die relative density $\rho_{c}$ increases as the lubricant concentration and mixing time increase. It is known that inter-particle bonding strength and inter-particle and tooling-particle friction decrease with an increase in lubrication [47,48]; hence, it is expected to observe that the packing jams at a higher density, and the compaction force decreases with an increase in lubrication. Furthermore, it is worth noting that while the effects of lubrication on bulk density reach a plateau at small mixing times, the parameters 1/b and $\rho_{c}$ vary at mixing times in the 15–25 min range. This shows that effects of mixing time can be seen downstream in the tablet press (e.g., changes in the drug release rate [9]) although this is not seen right after mixing (e.g., no effects on die-filling efficacy or even compaction force).

The lack of dependency of blends with colloidal silica to glidant concentration and mixing time can be understood in connection to the primary function served by the lubricant and the glidant. Magnesium stearate primarily coats particles reducing friction between particles and along die walls. Colloidal silica primarily offsets cohesion, improving the flowability of the blend. The former mechanism is at play during powder compaction, and the latter is not.

### 3.4. Elastic Recovery and Tablet Density

**Figure 8:**
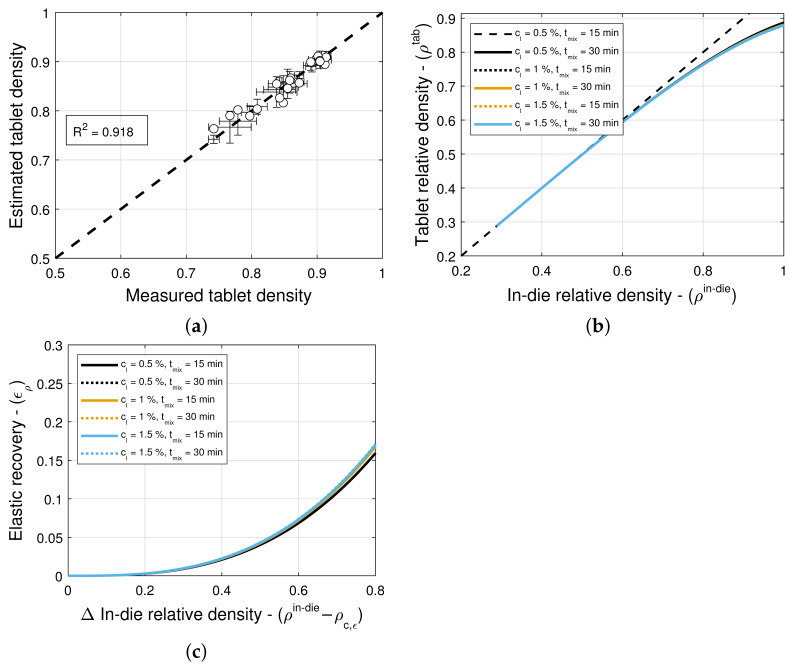
Tablet density and elastic recovery for blends with magnesium stearate (MgSt). (**a**) Goodness of the tablet density prediction. (**b**) Predictions of the tablet density model. (**c**) Predictions of elastic recovery.

**Figure 9:**
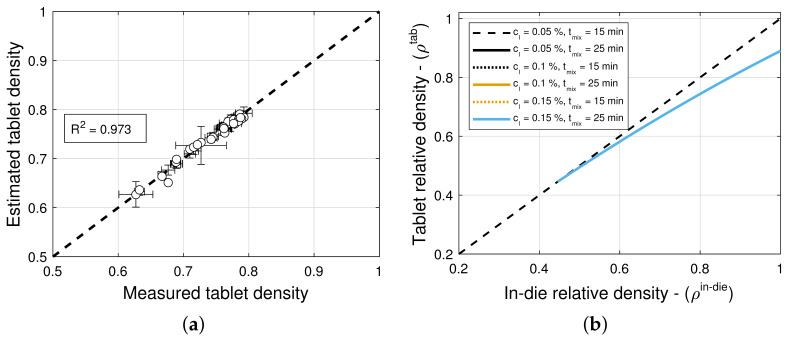
Tablet density and elastic recovery for blends with colloidal silica (CS). (**a**) Goodness of the tablet density prediction. (**b**) Predictions of the tablet density model.

The model selection and parameter estimation approach proposed in Section 2.3 identifies model (5,5) for blends with magnesium stearate, and model (9,9) for blends with colloidal silica as optimal within the model library F for $\epsilon_{0}$ and $\rho_{c,\epsilon }$ (see Table 13 and Table 14)—when restricting attention to only variants of Equation (16) for simplicity. Figure 8a and Figure 9a scatter plots of measured and estimated tablet density, demonstrating that the goodness of the tablet density prediction is high, with $R^{2}=0.918$ (Table 13) and $R^{2}=0.973$ (Table 14) for blends with magnesium stearate and colloidal silica, respectively. As it was the case for the compaction force model, the Akaike Information Criterion favors models that very closely fit the training data, i.e., $R^{2}\approx 1$, regardless of their simplicity. In this case, the model (5,5) for blends with magnesium stearate is overfitted, since the model parameters θ are excessively large in magnitude. Hence, one ought to select model (6,9) as optimal, with less model parameters that are bounded in magnitude.

Two common approaches that may be used to automate the selection of models with bounded parameter values are by enforcing bound constraints and applying regularization techniques. Here, the constraints on model parameter values were chosen only based on mechanistic interpretation limits. Similarly, we did not use off-the-shelf regularization techniques such as Ridge regularization, which calculates the L2 norm of parameter values, since the parameter values have mechanistic interpretations. Therefore, we used a post hoc model selection method. The development of better model evaluation metrics that account for large values of such parameters is ongoing work. Here, we raise the issue as it was encountered in this work, and we call for a resolution in future work.

Figure 8b and Figure 9b show predictions of the models for tablets formed with blends with different lubricant (glidant) concentrations and different mixing times. It is evident from the figures that these processing conditions have little to no influence on the relationship between in-die $\rho^{\mathrm{in}-\mathrm{die}}$ and out-of-die $\rho^{\mathrm{tablet}}$ tablet relative densities. This is in agreement with the observation that model (9,9) is optimal for blends with colloidal silica, i.e., the elastic recovery $\epsilon_{\rho }$ is insensitive to the glidant in the blend for concentrations under 0.2% *w*/*w*. However, for blends with magnesium stearate, Figure 8c suggests that there is a weak dependency on the lubricant concentration and mixing time.

### 3.5. Tensile Strength

**Figure 10:**
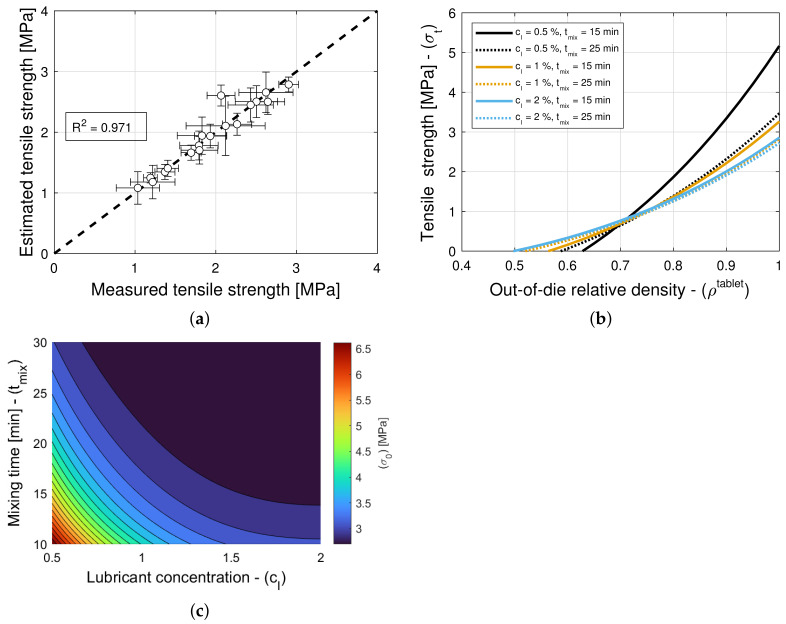
Tensile strength for blends with magnesium stearate (MgSt). (**a**) Goodness of the tensile strength prediction. (**b**) Predictions of the tensile strength model. (**c**) Contour plot of $\sigma_{0}$.

**Figure 11:**
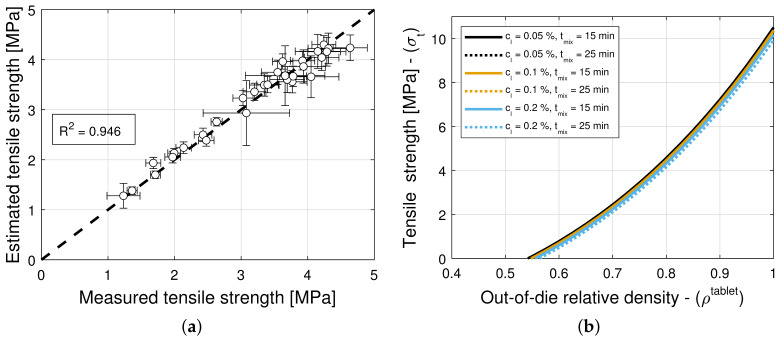
Tensile strength for blends with colloidal silica (CS). (**a**) Goodness of the tensile strength prediction. (**b**) Predictions of the tensile strength model.

The model selection and parameter estimation approach proposed in Section 2.3 identifies model (3,3) for blends with magnesium stearate, and model (9,5) for blends with colloidal silica as optimal within the model library F for $\sigma_{0}$ and $\rho_{c,\sigma_{t}}$ (see Table 15 and Table 16)—when restricting attention to only variants of Equation (16) for simplicity. Figure 10a and Figure 11a show scatter plots of measured and estimated tensile strength, demonstrating that the goodness of the tensile strength prediction is high, with $R^{2}=0.971$ (Table 15) and $R^{2}=0.946$ (Table 16) for blends with magnesium stearate and colloidal silica, respectively. As it was the case for the elastic recovery and tablet density model, the Akaike Information Criterion favors an overfitted model, namely (9,5) for blends with colloidal silica. Hence, model (8,8) ought to be selected instead, since it is the best ranked model with model parameters that are bounded in magnitude.

Figure 10b and Figure 11b show predictions of the models for tablets formed with blends with different lubricant (glidant) concentrations and different mixing times. It is evident from Figure 11b that the tablet tensile strength $\sigma_{t}$ is insensitive to the glidant in the blend for concentrations under 0.2% *w*/*w*. There is only a weak dependency for the smallest tablet density $\rho_{c,\sigma_{t}}$ at which a tablet with adequate strength is formed. This is in agreement with the primary function served by the glidant, i.e., it offsets cohesion, improving the flowability of the blend. In turn, this reduction in cohesion appears to slightly offset the tablet-bonding strength at low compaction levels but not at high deformation levels, at which the strength is $\sigma_{0}=10.7$ MPa regardless of the glidant conditions (see Table 6). In contrast, it is observed in Figure 10b that the tensile strength exhibits a strong dependency on lubrication conditions, as it has been reported in the literature (see, e.g., Razavi et al. [10]). The model captures the reduction in tensile strength and critical tablet density with an increasing lubricant concentration or mixing time. By way of example, Figure 10c shows a contour plot of $\sigma_{0}$. It is interesting to note that the tensile strength at no porosity $\sigma_{0}$ of tablets forms with blends without a lubricant and without a glidant are predicted to be very similar (cf. $\sigma_{0}=p_{4}=10.7$ MPa in Table 5 and $\sigma_{0}=p_{4}=9.44$ MPa in Table 6), despite being obtained from two different datasets.

## 4. Discussion

The ROMs presented above are based on product and process understanding, and their integration with lubricant or glidant mixing offers the opportunity to gain insight into these blending unit operations that have a dominant effect on each of the four stages of compaction. Hence, for each CPP and CQA, we examine which of the CPP|CMA (namely the concentration of lubricant or glidant, the mixing time or total shear imparted to the blend, the feed frame and turret speeds, and the filling height) appear consistently in the top five ranked models. Following Bachawala et al. [23], this process parameter or material attribute is said to have the most dominant effect or first-order effect on the observed CPP or CQA. The rank of the effect is degraded by one when the most dominant effect is observed to have a very small effect on the observed CPP or CQA (e.g., mixing time has a small effect on the elastic recovery of tablets formed using blends with magnesium stearate, despite being the most dominant process parameter). The first- and second-order effects on tableting CPPs and CQAs are listed in Table 17. For blends with magnesium stearate, the bulk density increases and the tablet tensile strength decreases with increasing lubricant concentration. The tensile strength of the tablets is also significantly sensitive to the mixing time, while the bulk density of the blend is not. The compaction force, in contrast, is more sensitive to the shear strain than the lubricant concentration, with a decreasing trend for increasing mixing time. The elastic recovery of the tablet is not affected by the lubrication of the blend. In contrast, the glidant concentration only inversely affects the bulk density of the blend. The compaction force, the tablet elastic recovery, and the tablet tensile strength are not affected by the presence of colloidal silica in the blend. Lastly, the filling efficacy of the die does mainly depend on the filling height, with blends containing the glidant being more sensitive than those containing the lubricant.

These effects have been quantified further by conducting a global sensitivity analysis of the integrated models using Sobol indices [49], which capture the sensitivity of model predictions to each input. The Sobol indices of all integrated lubricant and glidant mixing models are presented in Table 18. It is observed from this table that the results of the sensitivity analysis align with the description of the first- and second-order effects in Table 17.

It is worth noting that in continuous manufacturing operations, any changes in the glidant or lubricant concentration or in the total shear strain imparted during mixing will result in changes in the bulk density of the blend (Section 3.1). In turn, the tablet weight (Section 3.2), and the relative density at main compression (Equations (1) and (2)) will change, as well as the compaction force (Section 3.3), the tablet relative density (Section 3.4) and the tablet tensile strength (Section 3.5). For changes in magnesium stearate, the compaction force and the tablet tensile strength will experience compounding effects, since the tableting ROMs reveal that the model parameters also depend on the lubricant concentration and the total shear strain. The understanding and quantification of these effects are crucial for enabling real-time control under the quality-by-control framework. In the context of the quality-by-design framework, they are crucial for identifying the design space of the direct compression process, i.e., the operational regime that ensures product’s quality (see, e.g., [50]).

## 5. Conclusions

We have demonstrated the efficacy of the rational function-based approach proposed by Bachawala et al. [23] in integrating tableting reduced-order models with lubricant and glidant upstream mixing in pharmaceutical solid dosage form manufacturing processes. The approach automatically identifies the upstream critical material attributes and process parameters, such as the lubricant or glidant concentration and the mixing time, that describe the coupling to the first-order and second-order. It also systematically selects the mathematical form of such coupling using normalized bivariate rational functions, and it estimates model parameters. Glidants and lubricants are commonly used pharmaceutical excipients that not only enhance the flowability of powders and reduce the inter-particle friction, respectively, but they also negatively affect the tablet’s critical quality attributes, e.g., by reducing the tablet tensile strength or the drug release rate. Hence, the tableting reduced-order models developed in this work have enabled the quantification of these effects on each of the four stages of powder compaction, namely die filling, compaction, unloading, and ejection. Specifically, formulations composed of 10% by mass of acetaminophen, microcrystalline cellulose, and varying small concentrations of magnesium stearate or colloidal silica were mixed. Tablets with a wide range of relative densities were fabricated using a Natoli NP-400 tablet press and varying dosing positions and turret speeds. Next, for each CPP and CQA, we have examined which of the upstream critical material attributes and process parameters appear consistently in the top five ranked models in the library of rational functions, i.e., we have identified the most dominant effect or first-order effect on the observed CPP or CQA. At the fixed dosing position and in-die tablet thickness, changes in the glidant or lubricant concentration or in the total shear strain imparted during mixing directly affect the tablet weight and, thus, the relative density at main compression. Changes in the latter, in turn, impact the compaction force, the tablet relative density, and the tablet tensile strength. Variations in magnesium stearate introduce further compounding effects. Since the lubricant reduces the inter-particle friction and weakens inter-particle solid bridges, the compaction force and the tablet tensile strength decrease with an increasing lubricant concentration and mixing time. Our approach quantifies these effects, and it is based on product and process understanding. Therefore, the approach is essential to enabling the end-to-end integration, control, and optimization of blending and tableting processes. However, technologies for measuring at-line these CMAs and CPPs are in the early stages of development. Hence, research directions worth exploring include NIR and Raman monitoring techniques, hybrid mechanistic/machine learning soft sensors, and model-based data reconciliation by using redundant PAT sensor networks—each contributing to the development of reliable methods for indirectly measuring very low MgSt/CS concentrations and the total shear imparted to the blend. The models developed in this work can be used for off-line design, as shown by Shahab et al. [50]. Furthermore, direct/indirect real-time measurements of MgSt/CS concentrations would complement the implementation of moving horizon estimate–nonlinear model predictive control (MHE-NMPC) for a continuous tablet manufacturing line [16]. Another crucial research direction that must be explored is modeling the combined effect of lubricant and glidants in the same blend, since the models proposed in this work model their effects separately. However, the proposed methodology could be used as a starting point for such a combined model of CQAs and CPPs.

We have also challenged the efficacy of the rational function-based approach proposed by Bachawala et al. [23] in systematically selecting the mathematical form of the coupling between tableting and upstream unit operations. For coupling models with a very small number of parameters, as it is the case here when the effects from upstream operations are negligible, we have observed that the Akaike Information Criterion favors models that very closely fit the training data, i.e., $R^{2}\approx 1$, regardless of their simplicity. We have also observed that it favors overfitted models, since their model parameters θ are excessively large in magnitude. The investigation and application of an AIC with higher-order estimates of information loss, as well as approaches that automatically exclude overfitted models, are research directions worthwhile exploring. The effective detection of multicollinearity in the coupling model may be a promising step in the right direction, if beyond the scope of this work.

## Figures and Tables

**Table 1:** Normalization of the upstream lubricant or glidant mixing CPP|CMA.

CPP|CMA	Lower and Upper Bounds	Normalized CPP|CMA
Lubricant $c_{\mathrm{MgSt}}$ concentration	0 ; $c_{\mathrm{MgSt}}^{\max}=2\%$ *w*/*w*	$X={\left(\frac{c_{\mathrm{MgSt}}}{c_{\mathrm{MgSt}}^{\max}-c_{\mathrm{MgSt}}}\right)}^{r_{X}}$
Glidant $c_{\mathrm{CS}}$ concentration	0 ; $c_{\mathrm{CS}}^{\max}=0.2\%$ *w*/*w*	$X={\left(\frac{c_{\mathrm{CS}}}{c_{\mathrm{CS}}^{\max}-c_{\mathrm{CS}}}\right)}^{r_{X}}$
Shear strain or mixing time $\gamma_{\mathrm{mix}}\propto t_{\mathrm{mix}}$	0 ; $\gamma_{\mathrm{mix}}^{\max}\propto t_{\mathrm{mix}}^{\max}=60$ min	$Y={\left(\frac{\gamma_{\mathrm{mix}}}{\gamma_{\mathrm{mix}}^{\max}-\gamma_{\mathrm{mix}}}\right)}^{r_{Y}}={\left(\frac{t_{\mathrm{mix}}}{t_{\mathrm{mix}}^{\max}-t_{\mathrm{mix}}}\right)}^{r_{Y}}$

**Table 2:** ROM parameters ξ modeled as bivariate rational functions of *X* and *Y*.

	Bulk Density	Compaction Force	Elastic Recovery	Tensile Strength
M	$\rho_{b}$	$F_{\mathrm{punch}}$	$\epsilon_{\rho }$	$\sigma_{t}$
ξ	$\rho_{b}\left(X,Y\right)$	a(X,Y) or 1/b(X,Y)	$\epsilon_{0}\left(X,Y\right)$	$\sigma_{0}\left(X,Y\right)$
		$\rho_{c}\left(X,Y\right)$	$\rho_{c,\epsilon }\left(X,Y\right)$	$\rho_{c,\sigma_{t}}\left(X,Y\right)$

**Table 3:** Constrained variants of the normalized bivariate rational function f(X,Y) in Equations (16) and (18).

Model Number	Rational Function	No. of Parameters
1	Equation (16): f(X,Y)	7
2	Equation (16): $r_{X}=r_{Y}=1$	5
3	Equation (16): $q_{3}=0$	6
4	Equation (16): $q_{3}=0$, $r_{X}=r_{Y}=1$	4
5	Equation (16): $q_{2}=0$	6
6	Equation (16): $q_{2}=0$, $r_{X}=r_{Y}=1$	4
7	$q_{2}=q_{3}=0$ (or $q_{5}=q_{6}=0$)	5
8	$q_{2}=q_{3}=0$ (or $q_{5}=q_{6}=0$), $r_{X}=r_{Y}=1$	3
9	Constant—$f\left(X,Y\right)=p_{4},r_{X}=r_{Y}=1$	1
10	Equation (18): f(X,Y)	7
11	Equation (18): $r_{X}=r_{Y}=1$	5
12	Equation (18): $q_{6}=0$	6
13	Equation (18): $q_{6}=0$, $r_{X}=r_{Y}=1$	4
14	Equation (18): $q_{5}=0$	6
15	Equation (18): $q_{5}=0$, $r_{X}=r_{Y}=1$	4

**Table 4:** Model library F: pairs of constrained variants of bivariate rational functions used by compaction force, elastic recovery, and tensile strength.

Function Variant	1	2	3	4	5	6	7	8	9	10	11	12	13	14	15
1	✔								✔						
2		✔							✔						
3			✔						✔						
4				✔					✔						
5					✔				✔						
6						✔			✔						
7							✔		✔						
8								✔	✔						
9	✔	✔	✔	✔	✔	✔	✔	✔	✔	✔	✔	✔	✔	✔	✔
10									✔	✔					
11									✔		✔				
12									✔			✔			
13									✔				✔		
14									✔					✔	
15									✔						✔

**Table 5:** Summary of the best lubricant models for tablet CQAs and CPPs with the corresponding parameters and 95% confidence intervals obtained from bootstrapping method [38].

$X={\left(\frac{c_{\mathrm{MgSt}}}{c_{\mathrm{MgSt}}^{\max}-c_{\mathrm{MgSt}}}\right)}^{r_{X}}$ , $Y={\left(\frac{t_{\mathrm{mix}}}{t_{\mathrm{mix}}^{\max}-t_{\mathrm{mix}}}\right)}^{r_{Y}}$	$c_{\mathrm{MgSt}}^{\max}=2\%$ , $t_{\mathrm{mix}}^{\max}=60\;\min$	
**Bulk density**		
$f\left(X,Y\right)=\frac{p_{1}XY+p_{4}\left(q_{5}X^{2}+1\right)}{q_{1}XY+q_{5}X^{2}+1}\rho_{t}\left(c_{\mathrm{MgSt}}\right)$	$r_{X}=1$ ;	$r_{Y}=1$
	$p_{1}=14.21\pm 4.31$ ;	$q_{1}=51.58\pm 15.88$
	$p_{4}=0.17\pm 0.007$ ;	$q_{5}=0.17\pm 0.032$
**Weight**	$W=\eta \rho_{b}V^{\mathrm{fill}}$	
$\eta =\xi_{2}\frac{n_{F}}{n_{T}}+\xi_{3}\frac{H^{\mathrm{fill}}}{D}+\xi_{4}{\left(\frac{t^{\mathrm{fill}}}{D}\right)}^{2}$	$\xi_{2}=0.076\pm 0.007$ ; $\xi_{3}=1.281\pm 0.024$ ; $\xi_{4}=-0.533\pm 0.015$	
**Compaction force**	$F_{\mathrm{punch}}=\frac{\pi D^{2}\left(\rho^{\mathrm{in}-\mathrm{die}}-\rho_{c}\right)}{4b\left(\rho^{\mathrm{in}-\mathrm{die}}\left(a-1\right)+\rho_{c}\right)}$	
	$r_{X}=0.85\pm 0.35$ ; $r_{Y}=8.76\pm 0.65$	
	a=0.735±0.013	
$\frac{1}{b}=\frac{p_{1}XY+p_{4}\left(q_{2}X+1\right)}{q_{1}XY+q_{2}X+1}$	$p_{1}=0.00174\pm 0.02\;\mathrm{MPa}$ ;	$q_{1}=220.6\pm 67.67$
	$p_{4}=0.033\pm 0.013\;\mathrm{MPa}$ ;	$q_{2}=0.0034+0.05$
$\rho_{c}=\frac{p_{1}XY+p_{4}\left(q_{2}X+1\right)}{q_{1}XY+q_{2}X+1}$	$p_{1}=264.2\pm 78.44$ ;	$q_{1}=915.4\pm 360.52$
	$p_{4}=0.365\pm 0.02$ ;	$q_{2}=1.02\pm 0.44$
**Elastic recovery**	$\epsilon_{\rho }=\epsilon_{0}{\left(\frac{\rho^{\mathrm{in}}-\mathrm{die}-\rho_{c,\epsilon }}{1-\rho_{c,\epsilon }}\right)}^{n}$	
	$r_{X}=1$ ; $r_{Y}=1$	
	n=2.928±0.027	
$\epsilon_{0}=\frac{p_{1}XY+p_{4}\left(q_{3}Y+1\right)}{q_{1}XY+q_{3}Y+1}$	$p_{1}=14.29\pm 1.64$ ;	$q_{1}=117.11\pm 13.54$
	$p_{4}=9.32\times 10^{-9}\pm 0.012$ ;	$q_{3}=4.99\times 10^{-7}\pm 6.146$
$\rho_{c,\epsilon }=p_{4}$	$p_{4}=0.288\pm 0.007$	
**Tensile strength**	$\sigma_{t}=\sigma_{0}\left[1-\left(\frac{1-\rho^{\mathrm{tablet}}}{1-\rho_{c,\sigma_{t}}}\right)e^{\left(\rho^{\mathrm{tablet}}-\rho_{c,\sigma_{t}}\right)}\right]$	
	$r_{X}=1.92\pm 0.071$ ; $r_{Y}=1.94\pm 0.099$	
$\sigma_{0}=\frac{p_{1}XY+p_{4}\left(q_{2}X+1\right)}{q_{1}XY+q_{2}X+1}$	$p_{1}=335.0\pm 81.23\;\mathrm{MPa}$ ;	$q_{1}=125.1\pm 23.96$
	$p_{4}=9.44\pm 0.13\;\mathrm{MPa}$ ;	$q_{2}=0.40\pm 0.10$
$\rho_{c,\sigma_{t}}=\frac{p_{1}XY+p_{4}\left(q_{2}X+1\right)}{q_{1}XY+q_{2}X+1}$	$p_{1}=5.40\pm 1.58$ ;	$q_{1}=10.85\pm 4.28$
	$p_{4}=0.649\pm 0.005$ ;	$q_{2}=1.77\times 10^{-7}\pm 0.044$

**Table 6:** Summary of the best glidant models for tablet CQAs and CPPs with the corresponding parameters and 95% confidence intervals obtained from bootstrapping method [38].

$X={\left(\frac{c_{\mathrm{CS}}}{c_{\mathrm{CS}}^{\max}-c_{\mathrm{CS}}}\right)}^{r_{X}}$ , $Y={\left(\frac{t_{\mathrm{mix}}}{t_{\mathrm{mix}}^{\max}-t_{\mathrm{mix}}}\right)}^{r_{Y}}$	$c_{\mathrm{CS}}^{\max}=0.2\%$ , $t_{\mathrm{mix}}^{\max}=60\;\min$	
**Bulk density**		
$\rho_{b}=\frac{p_{1}XY+p_{4}}{q_{1}XY+1}\rho_{t}\left(c_{\mathrm{CS}}\right)$	$r_{X}=0.325\pm 0.75$ ;	$r_{Y}=0.222\pm 1.09$
	$p_{1}=0.279\pm 6.09$ ;	$q_{1}=0.566\pm 81.14$
	$p_{4}=0.242\pm 0.07$ ;	
**Weight**	$W=\eta \rho_{b}V^{\mathrm{fill}}$	
$\eta =\xi_{1}+\xi_{2}\frac{n_{F}}{n_{T}}+\xi_{3}\frac{t^{\mathrm{fill}}}{D}+\xi_{4}{\left(\frac{t^{\mathrm{fill}}}{D}\right)}^{2}$	$\xi_{1}=0.0862\pm 0.044$ ;	$\xi_{2}=-0.0472\pm 0.003$ ;
	$\xi_{3}=2.1036\pm 0.075$ ;	$\xi_{4}=-1.1375\pm 0.032$ ;
**Compaction force**	$F_{\mathrm{punch}}=\frac{\pi D^{2}\left(\rho^{\mathrm{in}-\mathrm{die}}-\rho_{c}\right)}{4b\left(\rho^{\mathrm{in}-\mathrm{die}}\left(a-1\right)+\rho_{c}\right)}$	
	$r_{X}=1.0$ ; $r_{Y}=1.0$	
	a=0.824±0.001	
$\frac{1}{b}=p_{4}$	$p_{4}=0.0104\pm 0.003\;\mathrm{MPa}$	
$\rho_{c}=p_{4}$	$p_{4}=0.1977\pm 0.006$	
**Elastic recovery**	$\epsilon_{\rho }=\epsilon_{0}{\left(\frac{\rho^{\mathrm{in}}-\mathrm{die}-\rho_{c,\epsilon }}{1-\rho_{c,\epsilon }}\right)}^{n}$	
	$r_{X}=1.0$ ; $r_{Y}=1.0$	
	n=1.0	
$\epsilon_{0}=p_{4}$	$p_{4}=0.1098\pm 0.0004$	
$\rho_{c,\epsilon }=p_{4}$	$p_{4}=0.446\pm 0.0039$	
**Tensile strength**	$\sigma_{t}=\sigma_{0}\left[1-\left(\frac{1-\rho^{\mathrm{tablet}}}{1-\rho_{c,\sigma_{t}}}\right)e^{\left(\rho^{\mathrm{tablet}}-\rho_{c,\sigma_{t}}\right)}\right]$	
	$r_{X}=1$ ; $r_{Y}=1$	
$\sigma_{0}=\frac{p_{1}XY+p_{4}}{q_{1}XY+1}$	$p_{1}=161.0\pm 21.94\;\mathrm{MPa}$ ;	$q_{1}=17.18\pm 2.25$
	$p_{4}=10.7\pm 0.05\;\mathrm{MPa}$ ;	
$\rho_{c,\sigma_{t}}=\frac{p_{1}XY+p_{4}}{q_{1}XY+1}$	$p_{1}=13.80\pm 4.62$ ;	$q_{1}=23.80\pm 8.10$
	$p_{4}=0.53\pm 0.001$ ;	

**Table 7:** Top five best models within the model library F for bulk density $\rho_{b}$ of blends with magnesium stearate (MgSt)—see Table 3.

$\rho_{b}\left(X,Y\right)$	$N_{p}$	SSE	$R^{2}$	AIC
13	4	1359	0.700	92.37
11	5	1359	0.700	94.37
8	3	1677	0.629	94.58
12	6	1256	0.722	94.80
4	4	1634	0.639	96.06

**Table 8:** Top five best models within the model library F for bulk density $\rho_{b}$ of blends with colloidal silica (CS)—see Table 3.

$\rho_{b}\left(X,Y\right)$	$N_{p}$	SSE	$R^{2}$	AIC
7	5	563	0.828	96.58
5	6	563	0.828	96.70
3	6	563	0.828	97.91
1	7	563	0.828	98.05
8	3	838	0.744	98.70

**Table 9:** Top five best models for filling efficacy coefficient η of blends with magnesium stearate (MgSt).

$\eta (n_{F}/n_{T},t^{\mathrm{fill}}/D)$	$N_{p}$	$R^{2}$	AIC
Equation (23) with $\xi_{1}=\xi_{5}=0$	3	0.676	110.4
Equation (23) with $\xi_{1}=0$	4	0.702	110.7
Equation (23)	5	0.720	111.5
Equation (21)	2	0.582	113.5
Equation (23) with $\xi_{5}=0$	4	0.652	113.9

**Table 10:** Top five best models for filling efficacy coefficient η of blends with colloidal silica (CS).

$\eta (n_{F}/n_{T},t^{\mathrm{fill}}/D)$	$N_{p}$	$R^{2}$	AIC
Equation (23) with $\xi_{5}=0$	4	0.938	69.6
Equation (23) with $\xi_{1}=0$	4	0.938	69.6
Equation (23)	5	0.939	71.6
Equation (23) with $\xi_{1}=\xi_{5}=0$	3	0.919	75.6
Equation (21)	2	0.520	125.1

**Table 11:** Top five best models within the model library F for compaction force model parameters *a*, 1/b, and $\rho_{c}$ of blends with magnesium stearate (MgSt)—see Table 3 and Table 4.

a(X,Y)	1/b(X,Y)	$\rho_{c}\left(X,Y\right)$	$N_{p}$	SSE	$R^{2}$	AIC
9	3	3	11	57.32	0.977	36.39
3	9	3	11	75.88	0.970	47.61
1	9	1	13	74.22	0.971	50.73
9	5	5	11	83.02	0.967	51.21
9	1	1	13	88.61	0.965	57.81

**Table 12:** Top five best models within the model library F for compaction force model parameters *a*, 1/b, and $\rho_{c}$ of blends with colloidal silica (CS)—see Table 3 and Table 4.

a(X,Y)	1/b(X,Y)	$\rho_{c}\left(X,Y\right)$	$N_{p}$	SSE	$R^{2}$	AIC
6	9	6	9	2.244	0.992	−179.2
8	9	8	7	2.750	0.990	−171.0
9	9	9	3	3.156	0.988	−170.7
9	8	8	7	2.762	0.990	−170.7
8	9	9	5	3.096	0.989	−167.9

**Table 13:** Top five best models within the model library F for elastic recovery model parameters $\epsilon_{0}$ and $\rho_{c,\epsilon }$ of blends with magnesium stearate (MgSt)—see Table 3 and Table 4.

$\epsilon_{0}\left(X,Y\right)$	$\rho_{c,\epsilon }\left(X,Y\right)$	$N_{p}$	SSE	$R^{2}$	AIC	Note
5	5	11	0.0018	0.964	−163.8	Overfitting (excessively large θ)
6	9	6	0.0042	0.918	−157.5	Best model
7	9	7	0.0038	0.926	−157.4	
2	9	7	0.0040	0.921	−156.4	
9	8	5	0.0050	0.901	−155.7	

**Table 14:** Top five best models within the model library F for elastic recovery model parameters $\epsilon_{0}$ and $\rho_{c,\epsilon }$ of blends with colloidal silica (CS)—see Table 3 and Table 4.

$\epsilon_{0}\left(X,Y\right)$	$\rho_{c,\epsilon }\left(X,Y\right)$	$N_{p}$	SSE	$R^{2}$	AIC
9	9	3	0.002	0.973	−286
8	9	5	0.002	0.974	−283
9	8	5	0.002	0.974	−283
9	9	5	0.002	0.973	−282
4	9	6	0.002	0.975	−282

**Table 15:** Top five best models within the model library F for tensile strength model parameters $\sigma_{0}$ and $\rho_{c,\sigma_{t}}$ of blends with magnesium stearate (MgSt)—see Table 3 and Table 4.

$\sigma_{0}\left(X,Y\right)$	$\rho_{c,\sigma_{t}}\left(X,Y\right)$	$N_{p}$	SSE	$R^{2}$	AIC
3	3	10	20.19	0.971	−1845
1	1	12	20.18	0.971	−1841
7	7	8	21.36	0.969	−1818
5	5	10	21.22	0.969	−1817
3	9	7	23.43	0.966	−1768

**Table 16:** Top six best models within the model library F for tensile strength model parameters $\sigma_{0}$ and $\rho_{c,\sigma_{t}}$ of blends with colloidal silica (CS)—see Table 3 and Table 4.

$\sigma_{0}\left(X,Y\right)$	$\rho_{c,\sigma_{t}}\left(X,Y\right)$	$N_{p}$	SSE	$R^{2}$	AIC	Note
9	5	7	70.52	0.951	−4374	Overfitting (excessively large θ)
9	7	6	71.72	0.951	−4351	Overfitting (excessively large θ)
5	5	10	73.32	0.949	−4311	Overfitting (excessively large θ)
1	1	12	73.37	0.949	−4307	Overfitting (excessively large θ)
7	7	8	75.96	0.948	−4264	Overfitting (excessively large θ)
8	8	6	78.48	0.946	−4221	Best Model

**Table 17:** First- and second-order effects of lubricant and glidant mixing on tableting CPPs and CQAs.

Lubricant/Glidant	Tablet Weight		Compaction	Elastic	Tensile
Mixing	Bulk Density	Filling Efficacy	Force	Recovery	Strength
$c_{\mathrm{MgSt}}$	1st	—	2nd	—	1st
$\gamma_{\mathrm{mix}}$	2nd	—	1st	2nd	2nd
$n_{F}/n_{T}$	—	2nd	—	—	—
$t^{\mathrm{fill}}/D$	—	1st	—	—	—
$c_{\mathrm{CS}}$	1st	—	—	—	—
$\gamma_{\mathrm{mix}}$	2nd	—	—	—	2nd
$n_{F}/n_{T}$	—	2nd	—	—	—
$t^{\mathrm{fill}}/D$	—	1st	—	—	—

**Table 18:** Global sensitivity analysis of integrated models using Sobol indices. Values in each entry of the table denote sensitivity of each model to the corresponding input.

Lubricant/Glidant	Bulk	Tablet	Compaction	Elastic	Tensile
Mixing	Density	Weight	Force	Recovery	Strength
$c_{\mathrm{MgSt}}$	0.9073	-	0.0819	0.0508	0.1574
$\gamma_{\mathrm{mix}}$	0.0621	-	0.4199	0.0014	0.0115
$n_{F}/n_{T}$	-	0.0330	-	-	-
$t^{\mathrm{fill}}/D$	-	0.6538	-	-	-
$\rho_{b}$	-	0.3110	-	-	-
$\rho^{\mathrm{in}}-\mathrm{die}$	-	-	0.3124	0.9306	-
$\rho^{\mathrm{tablet}}$	-	-	-	-	0.6157
$c_{\mathrm{CS}}$	0.9579	-	0.0000	0.0000	0.0055
$\gamma_{\mathrm{mix}}$	0.0408	-	0.0000	0.0000	0.0003
$n_{F}/n_{T}$	-	0.0102	-	-	-
$t^{\mathrm{fill}}/D$	-	0.8383	-	-	-
$\rho_{b}$	-	0.1491	-	-	-
$\rho^{\mathrm{in}}-\mathrm{die}$	-	-	1.0000	1.0000	-
$\rho^{\mathrm{tablet}}$	-	-	-	-	0.9938

## Data Availability

The original contributions presented in this study are included in the article; any further inquiries can be directed to the corresponding authors.

## Abbreviations

**Symbol**	**Description**
$\rho^{\mathrm{pc}}$	Pre-compression relative density
$\rho^{\mathrm{in}-\mathrm{die}}$	Main-compression relative density
$\rho^{\mathrm{tablet}}$	Tablet relative density after elastic recovery
*W*	Tablet weight
$\rho_{t}$	True density
$V^{\mathrm{pc}|\mathrm{in}-\mathrm{die}|\mathrm{tablet}}$	Pre-compression/main-compression/tablet volume
*D*	Tablet diameter
$t^{\mathrm{pc}|\mathrm{in}-\mathrm{die}|\mathrm{tablet}}$	Pre-compression/main-compression/tablet thickness excluding the convex parts of the tablet
$\sigma_{t}$	Table tensile strength
*F*	Crushing force of the tablet (hardness)
$H^{\mathrm{tablet}}$	Tablet height
η	Die-filling efficacy coefficient
$F_{\mathrm{punch}}$	Kawakita’s compaction force
$\epsilon_{\rho }$	Elastic recovery
$\rho_{b}$	Bulk density
$V_{\mathrm{fill}}$	Fill volume
*h*	Cup depth
$\rho^{\mathrm{in}-\mathrm{die}}$	In-die tablet relative density
*a*	Kawakita parameter, degree of maximum compression
1/b	Kawakita parameter, pressure needed for the powder to reach half of the maximum volume reduction (a/2)
$\epsilon_{0}$	In-die elastic recovery at full compaction (zero porosity)
$\rho_{c,\epsilon }$	Smallest in-die tablet relative density at which elastic recovery is observed
*n*	Exponent
$\sigma_{0}$	Theoretical maximum tensile strength of a tablet (achieved when relative density is one)
$\rho_{c,\sigma_{t}}$	Smallest tablet relative density at which tablet with adequate strength is formed
$c_{\mathrm{CS}}$	Glidant concentration
$c_{\mathrm{MgSt}}$	Lubricant concentration
ξ	Reduced-order model parameters that depend on upstream CMAs/CQAs
M(ξ)	Tableting reduced-order model
θ	Reduced-order model parameters determined fro optimization
$c_{\mathrm{MgSt}}^{\max}$	Maximum glidant concentration
$c_{\mathrm{CS}}^{\max}$	Maximum lubricant concentration
$\gamma_{\mathrm{mix}}$	Total shear imparted to the blend
$t_{\mathrm{mix}}$	Total mixing time
$t_{\mathrm{mix}}^{\max}$	Maximum mixing time
*X*	Bivariate rational function variable corresponding to lubricant/glidant concentration
*Y*	Bivariate rational function variable corresponding to lubricant/glidant mixing time
f(X,Y)	Normalized bivariate rational function
$\left\{p_{i},q_{j},r_{X},r_{Y}\right\}$	Bivariate rational function parameters
F	Model library
$N_{p}$	Number of model parameters
M(θ)	Model prediction
E	Experimental data
*n*	Number of experimental data points
ϕ(X,Y)	Packing fraction of the granular system
$n_{T}$	Turret speed
$t^{\mathrm{fill}}$	Dosing position
$n_{F}$	Paddle or feed frame speed
η	Filling efficacy
$N_{d}$	Number of die stations in the tablet press
**Abbreviation**	**Description**
MgSt	Magnesium stearate
APIs	Active pharmaceutical ingredient particles
MCC	Microcrystalline cellulose
CQAs	Critical quality attributes
CPPs	Critical process parameters
CS	Colloidal silica
QbD	Quality by Design
QbC	Quality by Control
ROMs	Reduced-order models
CMAs	Critical material attributes
APAP	Acetaminophen
AIC	Akaike Information Criterion
